# Living in the waterfalls: A new species of *Trichomycterus* (Siluriformes: Trichomycteridae) from Tabay stream, Misiones, Argentina

**DOI:** 10.1371/journal.pone.0179594

**Published:** 2017-06-22

**Authors:** Guillermo Enrique Terán, Juliano Ferrer, Mauricio Benitez, Felipe Alonso, Gastón Aguilera, Juan Marcos Mirande

**Affiliations:** 1 Instituto de Vertebrados, Fundación Miguel Lillo, UEL–CONICET, San Miguel de Tucumán, Argentina; 2 Programa de Pós–Graduação em Biologia Animal, Departamento de Zoologia, Universidade Federal do Rio Grande do Sul, Porto Alegre, Rio Grande do Sul, Brazil; 3 Instituto de Biología Subtropical, UNaM–CONICET, Laboratorio de Genética Evolutiva, Posadas, Argentina; 4 Instituto de Bio y Geociencias del NOA (IBIGEO–CONICET), Rosario de Lerma, Salta, Argentina; SOUTHWEST UNIVERSITY, CHINA

## Abstract

A new species assigned to the genus *Trichomycterus* from the area of the waterfalls of Tabay stream, Paraná River basin, Misiones, Argentina, is described. *Trichomycterus ytororo* sp. nov. is distinguished from all other species in the genus by the presence of 31–35 dorsal procurrent caudal-fin rays and the combination of some external characters such as: coloration, number of pectoral–fin rays and pores of the laterosensory canals. The new taxon belongs to a presumably monophyletic group of species composed of *T*. *crassicaudatus*, *T*. *igobi*, and *T*. *stawiarski* based on the presence of 24 or more thickly ossified and rigid procurrent caudal-fin rays with a slender distal tip extending along the tips of at least ten neural spines.

## Introduction

Trichomycteridae is a monophyletic group comprising seven monophyletic subfamilies (Copionodontinae, Glanapteryginae, Sarcoglanidinae, Stegophilinae, Trichogeninae, Tridentinae, and Vandelliinae) and the recognized non–monophyletic subfamily Trichomycterinae as presently conceived [1,2,3,4,5]. Among the eight genera of Trichomycterinae, *Bullockia* Arratia, Chang, Menu-Marque & Rojas, *Eremophilus* Humboldt, *Hatcheria* Eigenmann, and *Rhizosomichthys* Miles, are monotypic based on autapomorphies; *Scleronema* Eigenmann is a monophyletic group including three psamophyly species; *Ituglanis* Costa & Bockmann and *Silvinichthys* Arratia were proposed to allocate species previously included in *Trichomycterus* Valenciennes; and lastly, *Trichomycterus* lacks any diagnostic character being demonstrably non-monophyletic assemblage of species [2,3,4,5].

*Trichomycterus* is currently the largest genus within Trichomycteridae, containing over 170 valid species [6], which are distributed in practically all South America, Costa Rica and Panamá in Central America [7], and even found in an offshore island in the Pacific Ocean [8]. Its geographical distribution ranges from lowlands of the Atlantic forest in southern Brazil, to mid-elevation streams on both sides of the Andean cordilleras, as well as the Patagonian region in Argentina [9]. Most species inhabit fast-flowing streams [10]. Their range of habitats is also remarkably diverse, including unusual freshwater habitats, such as sand-bottom streams, semipermanent pools, high-altitude Andean streams, and desolate creeks in Tierra del Fuego [11].

During recent samplings in northeastern Argentina we collected specimens of a remarkable new species of *Trichomycterus* from waterfalls in the Paraná River basin, which are described herein as a new species. The new taxon exhibits conspicuous characters that indicate a close relationship with *T*. *crassicaudatus* Wosiacki & de Pinna, *T*. *igobi* Wosiacki & de Pinna, and *T*. *stawiarski* (Miranda Ribeiro).

## Material and methods

Specimens were collected with hand nets at Tabay stream, Jardín América, Misiones, Argentina. Appropriate actions were taken to minimize pain or discomfort of fish. Specimens were euthanized by immersion in an anesthetic solution (0.1% 2-phenoxyethanol), and then fixed in a 4% formaldehyde for one week, washed in water for one day and transferred to a 70% ethanol solution for preservation. These steps are approved by the ethical use animals of Fundación Miguel Lillo (FML) that consider animal welfare laws. Field studies did not involve endangered species. Collection permit was granted by Ministerio de Ecología of Misiones (Disp 074/15). Measurements were taken point-to-point as linear distances with digital calipers to the nearest 0.1 mm. Number of vertebrae, ribs, odontodes, unsegmented dorsal- and anal-fin rays, and caudal-fin procurrent rays were taken from 11 radiographed specimens (rd) and two cleared and stained [12] specimens (c&s) whenever possible. Branchiostegal rays were counted in c&s specimens. The number of post-Weberian vertebrae includes the compound caudal centrum counted as a single element. Anteriormost unbranched fin rays are represented by lower case Roman numerals and branched fin rays by Arabic numerals. The musculature of the dorsolateral head was observed in one paratype (ms). Counst of the holotype are given in parentheses. Meristic and morphometric data, nomenclature in the osteology, myology and cephalic laterosensory system follows the most recent contributions in that sense [13,14,15,16]. The new taxon plus the putative close species *T*. *crassicaudatus*, *T*. *igobi*, and *T*. *stawiarski* are treated as *Trichomycterus stawiarski* group in the text. Species from the *Trichomycterus hasemani* group were not included in the comparisons considering their well-supported relationship with non-trichomycterinae taxa [2,17]. Institutional abbreviations: CI-FML (Colección ictiológica Fundación Miguel Lillo, San Miguel de Tucumán). LGEP (Laboratorio de Genética Evolutiva-Peces, Posadas). IBIGEO-I (Instituto de Bio y Geociencias del NOA- Ictiología, Salta, Argentina)

### Nomenclatural acts

The electronic edition of this work follows with the requirements of the International Code of Zoological Nomenclature, and hence the new names contained herein are available under that Code from the electronic edition of this article. This published work and the nomenclatural acts it contains have been registered in ZooBank, the online registration system for the ICZN. The ZooBank LSIDs (Life Science Identifiers) can be resolved and the associated information viewed through any standard web browser by appending the LSID to the prefix “http://zoobank.org/”. The LSID for this publication is: urn:lsid:zoobank.org:act:D4B81E7D-0D04-4907-852B-A36931736CBE

## Results

### *Trichomycterus ytororo*, new species

urn:lsid:zoobank.org:act:D4B81E7D-0D04-4907-852B-A36931736CBE

(Figs 1, 2 and 3)

**Fig 1:**
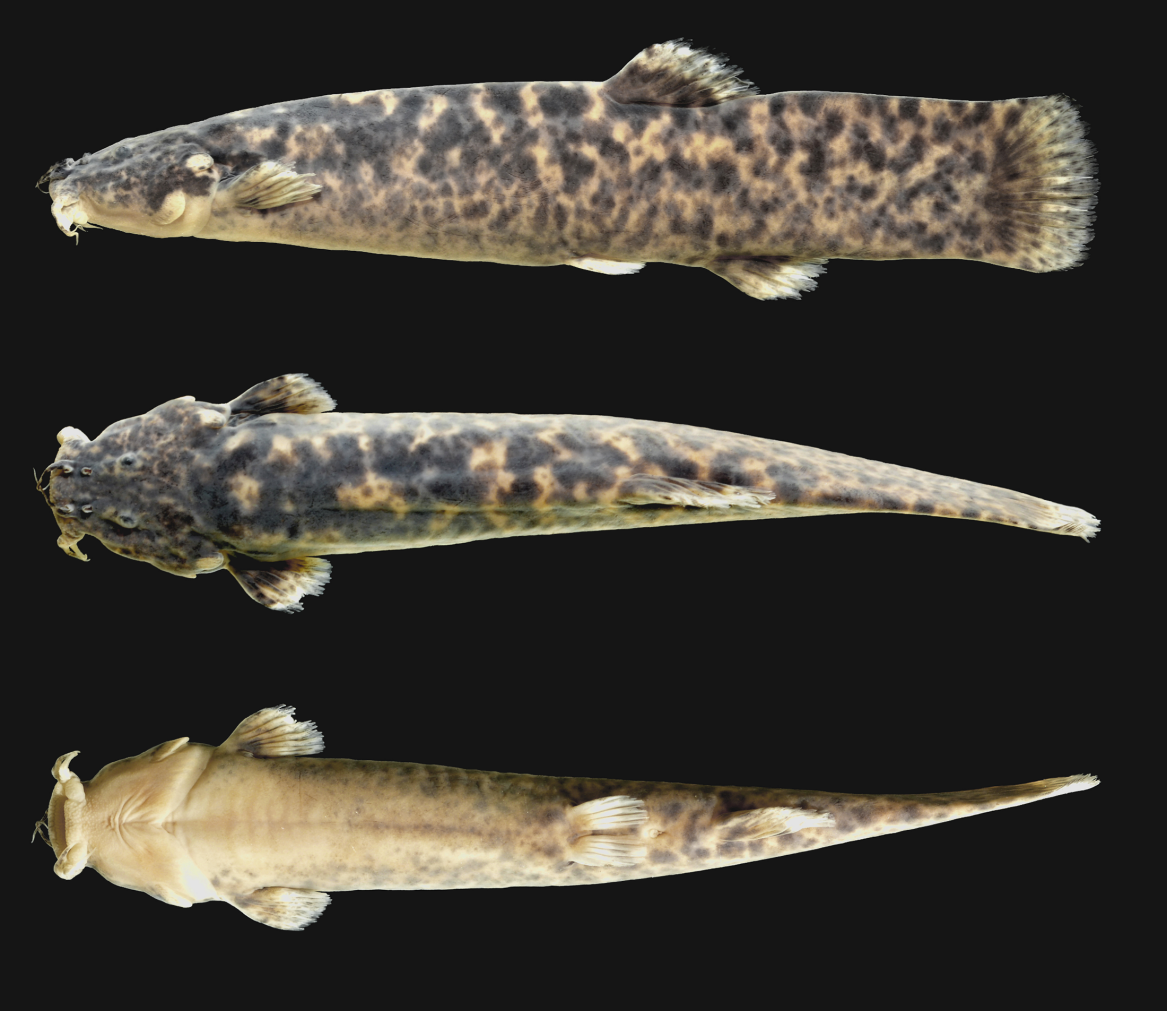
*Trichomycterus ytororo*, holotype, CI–FML 7240, 94.2 mm SL; Argentina, Misiones Province, Jardín América, Tabay stream, Paraná River basin.

**Fig 2:**
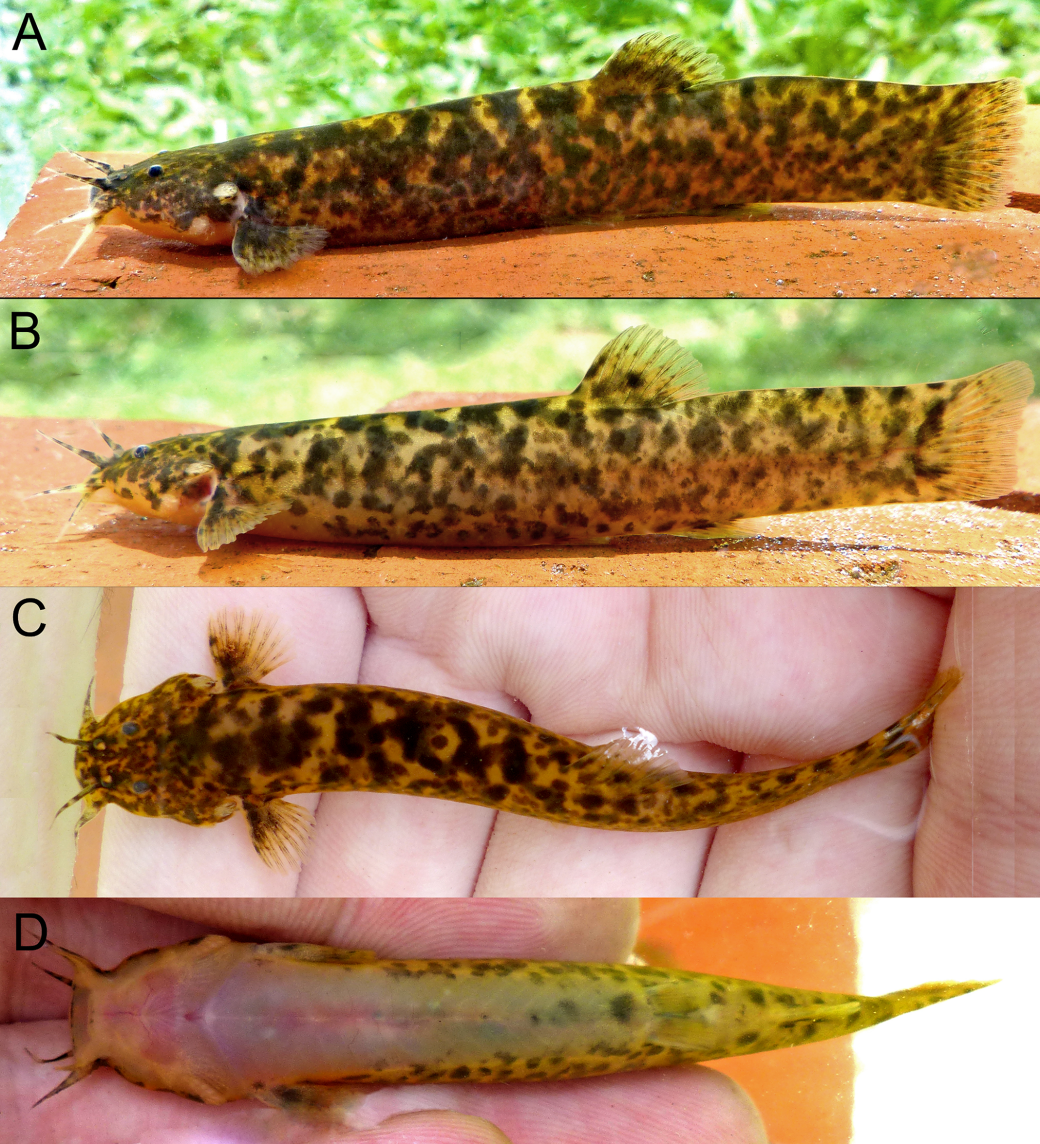
*Trichomycterus ytororo*, live specimens (A). holotype CI–FML 7241, 94.2 mm. (B–D). paratype CI–FML 7241, 60.9 mm SL. Argentina, Misiones, Jardín América, Tabay stream, Paraná River basin.

**Fig 3:**
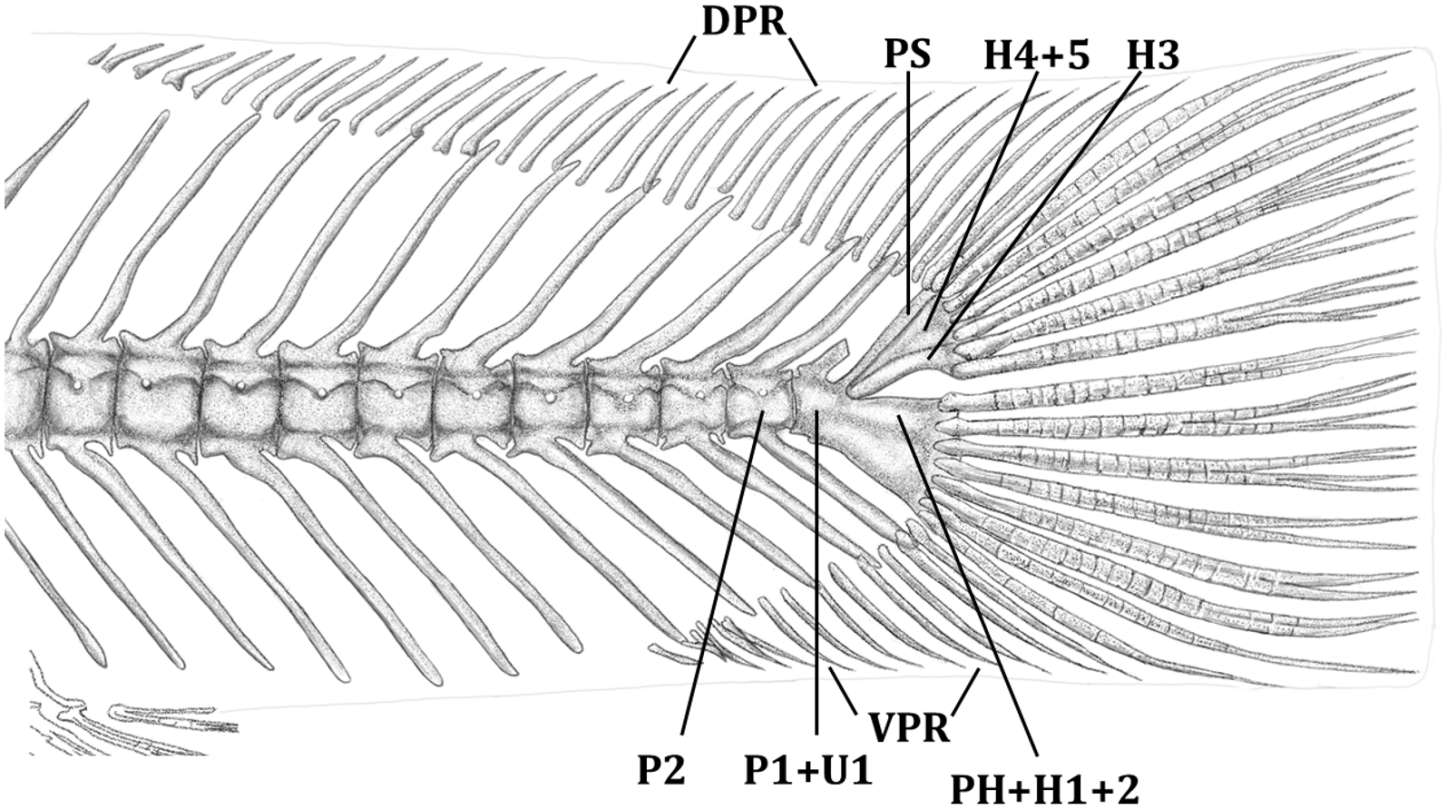
Left lateral view of the caudal peduncle of *Trichomycterus ytororo*. Paratype c&s specimen, CI–FML 7241, 67.3 mm SL. Abbreviations: DPR: dorsal procurrent caudal-fin rays, PH+H1+2 = lower caudal plate comprised by co-ossification of parhypural and hypurals 1 and 2; H4+5 = plate comprised by co-ossification of hypurals 4 and 5; pu1 + u1 = preural centrum 1 and ural centrum 1, pu2 = preural centrum 2; PS: pleurostile, VPR: ventral procurrent caudal-fin rays.

#### Holotype

CI–FML 7240 (rd), 94.2 mm SL, Argentina, Misiones, Jardín America, Tabay Waterfalls, Paraná River basin, 27°00’00”S 55°10’44”W, November 2016, G. E. Terán, M. Benitez, F. Alonso, G. Aguilera & J. M. Mirande.

#### Paratypes

All collected with holotype. CI–FML 7241, 67.3–103.8 mm SL, (8 rd, 1 c&s). LGEP 542, 4, 64.6–107.3 mm SL, (2 rd, 1 c&s, 1ms). IBIGEO-I 354, 3, 60.9–79.2 mm SL, (3 rd).

#### Diagnosis

*Trichomycterus ytororo* is distinguishable from all congeners by the presence of 31–35 dorsal caudal procurrent rays (Fig 3; vs. 29 or less). In addition, *Trichomycterus ytororo* exhibits two characters shared only by three congeners (Table 1; *T*. *crassicaudatus*, *T*. *igobi*, and *T*. *stawiarski*): the procurrent caudal-fin rays thickly ossified and rigid with a slender distal tip and the dorsal procurrent caudal-fin rays extending along the tips of 10–13 neural spines (Fig 3; vs. procurrent caudal-fin rays thin and flexible with the dorsal ones extending for less than eight neural spines tips). *Trichomycterus ytororo* can be further distinguished from *T*. *crassicaudatus*, *T*. *igobi*, and *T*. *stawiarski* by the number of branchiostegal rays (9 vs. 10–11). *Trichomycterus ytororo* can be further distinguished from *T*. *igobi* by the smaller head length (19.7–22.5% vs. 23.8–26.8% of SL), longest caudal peduncle (20.3–23.4% vs. 15.4–19.7% of SL) and 4–7 pores in the trunk canal of the laterosensory system (vs. two pores); from *T*. *crassicaudatus* by the caudal-fin distal margin rounded in adults (vs. forked), higher number of vertebrae (37–38 vs. 35–36), lower number of ventral procurrent caudal-fin rays (13 vs. 17–18), and the dorsal procurrent caudal-fin rays extending over the tips of 13 neural spines (vs. dorsal procurrent caudal-fin rays extending over the tips of 12 neural spines); and from *T*. *stawiarski* by the lower number of ventral procurrent caudal-fin rays (13 vs. 17), the higher number of pectoral-fin rays (i,7 vs. i,6), and lower number of vertebrae (37–38 vs. 39).

**Table 1:** Comparative characters among the species assigned to the *Trichomycterus stawiarski* group and its possible sister taxon *T*. *perkos*.

	*T*. *crassicaudatus*	*T*. *igobi*	*T*. *stawiarski*	*T*. *ytororo*, sp. nov.	*T*. *perkos*
Procurrent caudal–fin rays: thickness	thickly ossified and rigid	thickly ossified and rigid	thickly ossified and rigid	thickly ossified and rigid	thin and flexible
Dorsal procurrent caudal–fin rays: extension	over the tips of twelve neural spine	over the tips of at least ten neural spines	over the tips of thirteen neural spines	over the tips of thirteen neural spines	over the tips of less than 8 neural spines
Dorsal procurrent caudal–fin rays: number	24–26	24	27–29	31–35	14–17
Ventral procurrent caudal–fin rays: number	17–18	13	17	13	12–14
Branchiostegal rays: number	11	10–11	10	9	9–10
Caudal–fin distal margin in adults: shape	forked	truncate	truncate	rounded	rounded
Trunk canal pores: number	5–7	2	2–5	4–7	2
Pectoral–fin rays: number	i,7	i,7	i,6	i,7	i,5–6
Vertebrae: number	35–36	37	39	37–38	39–41
*Extensor tentaculi* muscle: origin	?	?	suspensorium and neurocranium	suspensorium and neurocranium	suspensorium

In addition to the above mentioned characters, *Trichomycterus ytororo* is distinguishable from congeners inhabiting the Paraná-Paraguay system by: 1) the number of pectoral-fin rays (i,7 vs. i,5 in *T*. *mboycy* Wosiacki & Garavello, *T*. *naipi* Wosiacki & Garavello, *T*. *piratymbara* Katz, Barbosa & Costa, *T*. *taroba* Wosiacki & Garavello; i,5–6 in *T*. *davisi* (Haseman), *T*. *perkos* Datovo, Carvalho & Ferrer; i,6 in *T*. *castroi* de Pinna, *T*. *maracaya* Bockmann & Sazima, *T*. *paolence* (Eigenmann), *T*. *papilliferus* Wosiacki & Garavello, *T*. *pauciradiatus* Alencar & Costa, *T*. *pirabitira* Barbosa & Azevedo-Santos, *T*. *septemradiatus* Katz, Barbosa & Costa; and i,8 in *T*. *boylei* (Nichols), *T*. *dali* Rizzato, Costa, Trajano & Bichuette, *T*. *therma* Fernández & Miranda). 2) First pectoral-fin ray not prolonged as filament (vs. first pectoral-fin ray prolonged as a long filament beyond the distal margin of the fin in *T*. *aguarague* Fernández & Osinaga and *T*. *taroba*. 3) The color pattern of lateral surface of body composed of black blotches over a pale yellow background extending to the mid-length of caudal fin (vs. lateral surface of body gray in *T*. *plumbeus* Wosiacki & Garavello and *T*. *papilliferus*; with well-defined spots in *T*. *iheringi* (Eigenmann); and proximal region of the caudal fin with a vertical unpigmented band in *T*. *diabolus* Bockmann, Casatti & de Pinna).

#### Description

Morphometric data of holotype and paratypes in Table 2. Holotype and paratypes illustrated in Figs 1–3.

**Table 2:** Morphometric data for *Trichomycterus ytororo*: holotype and paratypes (n = 12). SD: standard deviation. Range, Mean and SD include holotype.

	Holotype	Range	Mean	SD
Standard length (mm)	94.2	60.9–107.2		
**Percents of standart length**				
Head length	20.1	19.7–22.5	20.9	0.9
Predorsal length	60.5	60.2–63.9	61.9	1.0
Prepelvic length	55.2	54.9–58.5	56.4	1.1
Preanal length	69.9	67.7–73.8	71.3	1.6
Scapular girdle width	17.2	16.1–18.3	17.3	0.6
Trunk length	36.6	34.7–39.4	37.9	1.3
Pectoral–fin length	10.7	10.2–12.4	11.1	0.7
Pelvic–fin length	8.3	7.9–9.7	8.7	0.5
Distance between pelvic–fin base and anus	8.9	8.2–10.2	8.9	0.7
Caudal peduncle length	23.4	20.3–23.4	21.7	0.9
Caudal peduncle depth	18.2	15.6–18.2	17.1	0.8
Body depth	19.9	18.0–20.4	19.4	0.6
Length of dorsal–fin base	12.2	11.3–12.6	11.9	0.4
Length of anal–fin base	8.9	8.3–10.4	8.9	0.6
**Percents of head length**				
Head width	92.7	82.3–94.7	88.4	3.9
Nasal barbel length	29.2	22.5–33.9	29.0	3.3
Maxillary barbel length	32.2	23.1–33.8	30.0	3.5
Rictal barbel length	28.0	25–36.6	29.6	3.4
Snout length	42.4	36.5–42.6	40.7	2.0
Interorbital	21.2	19.1–24.4	21.8	1.6
Mouth width	32.4	26.8–34.5	31.8	2.2
Eye diameter	12.6	11.3–16.1	13.4	1.3
Supra–orbital pore distance	12.2	6.8–14	10.4	2.1

Body relative short, approximately rounded in transverse section at pectoral-fin level and laterally compressed towards tail. Dorsal profile from anterior margin of snout to dorsal-fin origin slightly convex; approximately straight from caudal peduncle to last caudal-fin rays. Ventral profile from tip of snout to pelvic fin slightly convex; straight from this point to last caudal-fin rays. Head relatively small, almost as long as wide, depressed from lateral view and trapezoidal from dorsal view. Snout broad, distal margin slightly convex from dorsal view. Dorsal and ventral profile of head slightly convex.

Eyes dorsally located on head but also visible from lateral view, anteroposteriorly elliptical, covered by thin and transparent skin. Anterior nostril smaller than eye, surrounded by fleshy flap of integument posterolaterally continuous with nasal barbel; distance between anterior nostrils slightly shorter than interorbital distance. Posterior nostril smaller than anterior one, partially surrounded anteriorly, laterally, and medially by thin flap of skin. Posterior nostrils more closely positioned to each other than anterior ones.

All barbels wider at bases and gradually narrowing towards tips. Nasal barbel extending to eye, not surpassing its posterior margin. Origin of nasal barbel on posterolateral margin of integument flap of anterior nostril. Maxillary and rictal barbels extending along cheek to vertical through eye, not reaching interopercular patch of odontodes. Origin of maxillary and rictal barbels on edge of lower and upper lips. Mouth wide, subterminal. Upper and lower lips fleshy, with similar size and bearing large and conspicuous papillae. Lower lip with lateral rounded fleshy lobes. Mental region with small papillae. Gill membranes united to isthmus anteriorly. Nine branchiostegal rays not visible through thick skin. Opercular and interopercular patches of odontodes with 17–19 and 30–34 conic and irregularly arranged odontodes, respectively. Odontodes surrounded by thick integument making smaller ones not visible through skin.

Laterosensory canals with simple (non-dendritic) tubes ending in single pores. Nasal and frontal canals of supraorbital line fused to each other, with three pores (s1, s3, and s6); one paratype with extra pores between pores s3 and s6 on both sides and one paratype with four pores between pores s3 and s6 on left side. Posterior segment of frontal, sphenotic, and otic canals fused to each other. Antorbital segment of infraorbital line absent. Sphenotic canal with two pores (i10 and i11). Otic, postotic and scapular canals with preoperculo-mandibular and pterotic branches short, each one with one associated pore. Trunk canal with 4–7 pores; fourth pore located at vertical through mid-length of pectoral fin.

Pectoral fin with i,7 rays (i,7) and rounded distal margin; third branched ray longest; first one not prolonged as filament. Dorsal fin located posteriorly to mid-length of body and inserted in a conspicuous concavity of dorsal surface of body. Dorsal fin with two or three unsegmented rays; ii,7 (ii,7) with distal margin rounded; first branched ray longest. Dorsal-fin pterygiophores eight, first one inserting anterior to neural spine of 16^th^ or 17^th^ vertebra (16^th^). Anal fin smaller in size than dorsal fin; anterior insertion located slightly posterior to dorsal-fin base. Anal fin with two unsegmented rays; i,5 rays (i,5) with distal margin rounded; first branched ray longest. Anal-fin pterygiophores six, first one inserting anterior to haemal spine of 21^st^ or 22^st^ vertebra (21^st^). Pelvic fin inserted anterior to vertical through dorsal-fin origin, not covering urogenital opening. Pelvic-fin rays i,4 (i,4); third and fourth rays longest; contralateral medial rays not overlapping in relaxed fins. Caudal fin with i,5 rays on upper hypural plate and i,6–7 rays on lower hypural plate (Fig 3) (i,6) with distal margin straight to slightly rounded. Smaller specimens (less than 67.3 mm SL) with anteriormost three caudal-fin rays of upper hypural plate slightly longer. Upper caudal plate with hypurals 4 and 5 fused together and hypural 3 autogenous; lower caudal plate with parahypural, hypurals 1 and 2 fused (Fig 3). Procurrent caudal-fin rays thickly ossified and rigid; 31–35 dorsally (holotype 34) and 13 ventrally (13) (Fig 3). Dorsal procurrent caudal-fin rays extending over tips of thirteen neural spines (13) and ventral procurrent caudal-fin rays extending dorsal to tips of five to six hemal spines (Fig 3) (holotype six). Vertebrae 37–38 (38). Ribs 11–12 pairs (11); first to sixth rib thicker.

#### Color in alcohol

Sides and dorsal surface of body and head with marbeled color pattern composed of black blotches irregular in shape and size, overlapped by superficial layer of small and less pigmented spots over pale yellow background, forming marbled color pattern (Fig 1). Blotches of dorsal surface of body and head larger, becoming smaller towards to ventrolateral region. Ventral surface of head and anteriormost portion of trunk pale yellow. Ventral surface of caudal peduncle region with inconspicuous and faint black blotches. Barbels with scattered black blotches dorsally and pale yellow ventrally. Opercular and interopercular patches of odontodes pale yellow. Pectoral, dorsal and anal fins with black blotches in proximal portion and whitish band distally. Pelvic fin hyaline with few inconspicuous black blotches. Caudal fin with black blotches in proximal portion becoming inconspicuous towards distal margin.

#### Color in life

Overall coloration of specimens in life similar to specimens after fixation with exception of brighter yellow background and distal portions of fins hyaline (Fig 2).

#### Ecological notes

**Fig 4:**
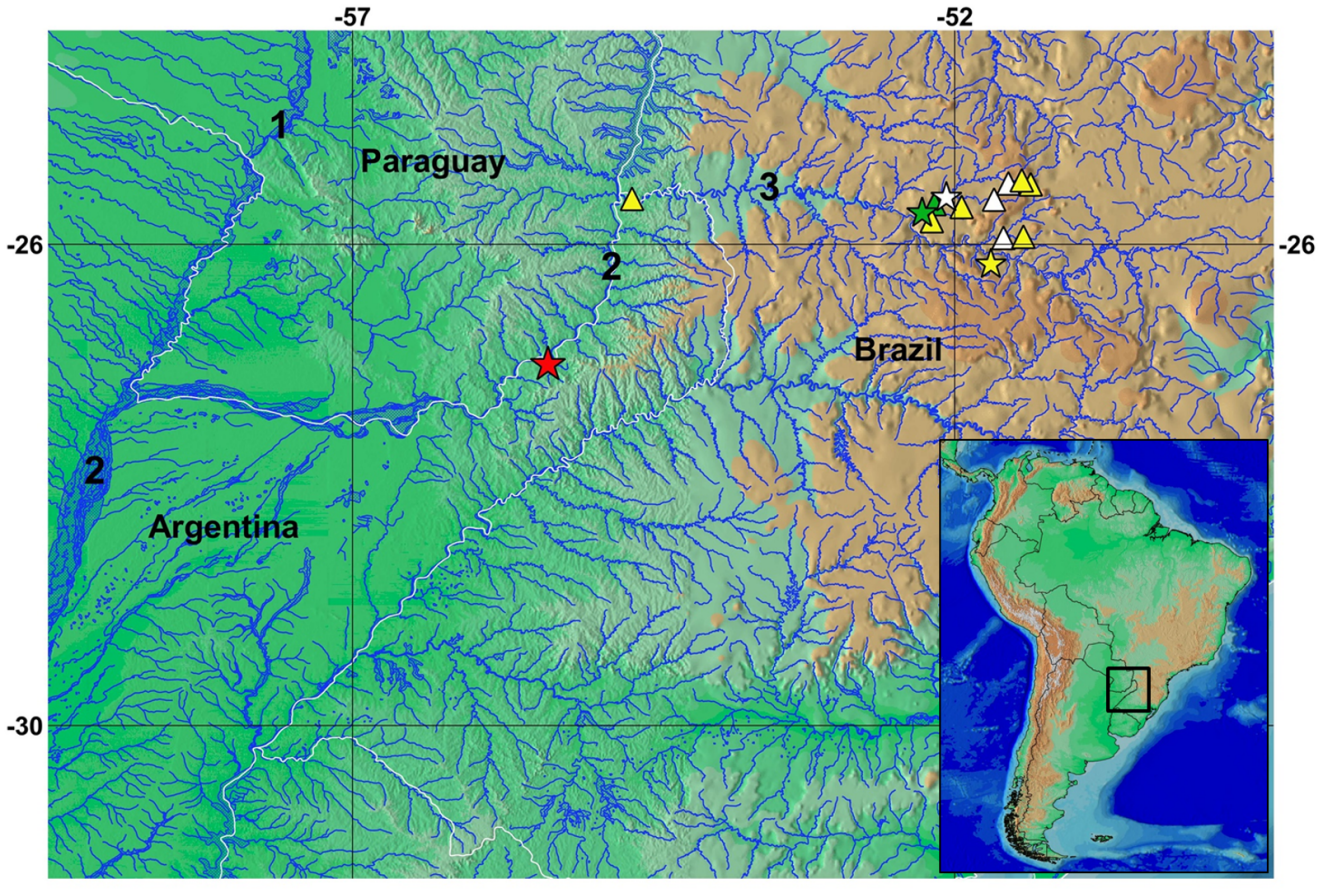
Geographic distribution of the species assigned to the *Trichomycterus stawiarski* group: *T*. *crassicaudatus* (green symbols), *T*. *igobi* (white symbols), *T*. *stawiarski* (yellow symbols) and *T*. *ytororo* (red symbol). Stars represent the type localities. Some triangles symbols represent more than one collection locality. Numbers 1, 2, 3 indicate the Paraguay, Paraná and Iguazú Rivers, respectively.

**Fig 5:**
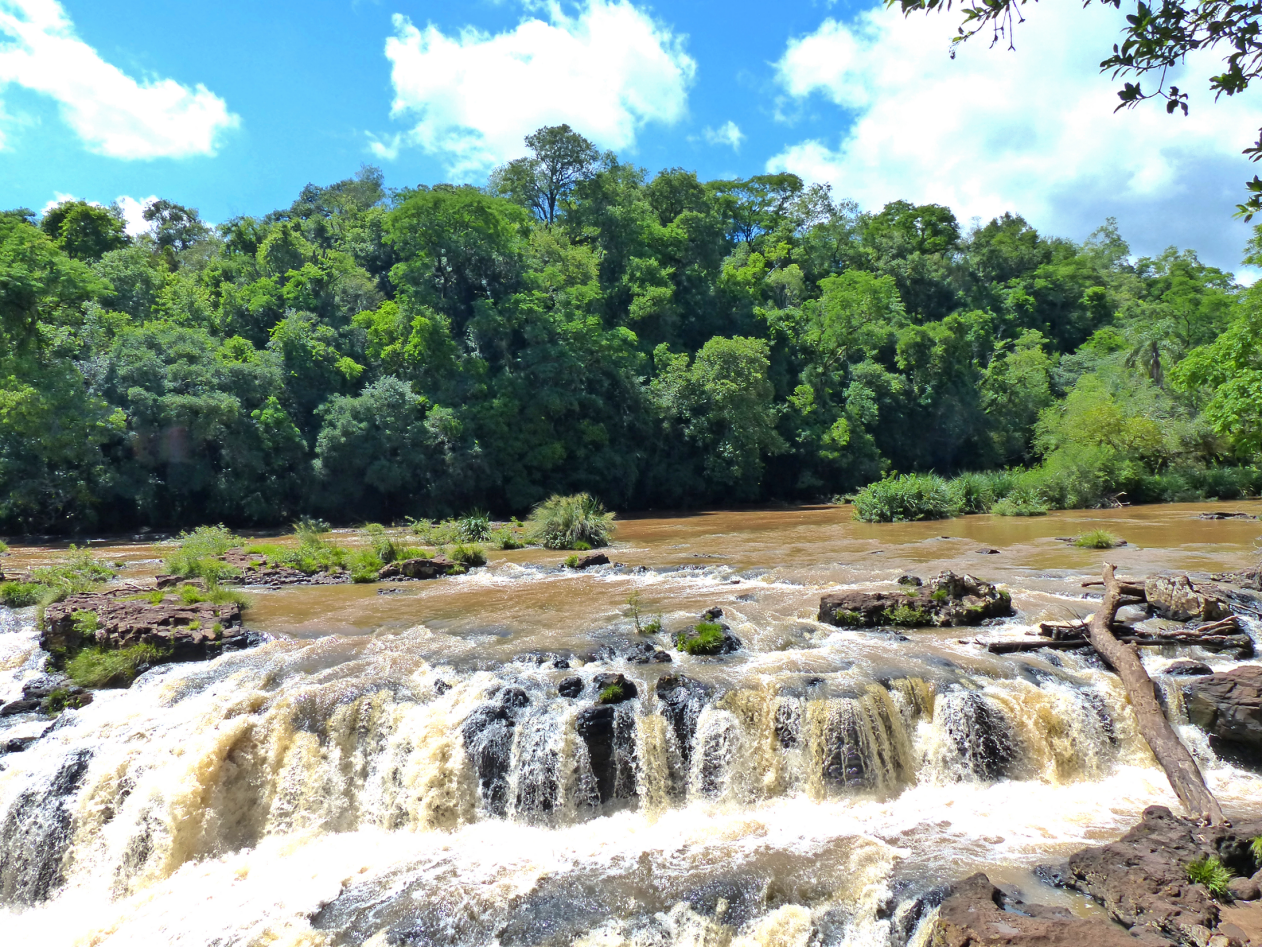
Type locality of *Trichomycterus ytororo*; Tabay waterfalls, Tabay stream, Jardín América, Misiones, Argentina.

The Tabay stream basin through 192 km from its headwaters at Campo Viera to its mouth on the Paraná River, at Jardín América (Fig 4). The stream bed is mainly composed of basaltic bedrock, in which sections with waterfalls and pools alternates all along its run. At the type locality (Tabay waterfall; Fig 5), the stream is surrounded by remnants of the Paranaense riparian forest, with its left margin degraded due to a camping site. This waterfall consists of three consecutive falls, the main one is 10m high and 20–50m wide, which drains into a narrow gorge. All specimens of *Trichomycterus ytororo* were captured above the waterfalls at shallow areas (about 1 meter depth or less) or in rapids witha predominantly rocky bottom and strong current. *Trichomycterus davisi* was the single congener recorded at the type locality, which was not collected syntopically with *T*. *ytororo*.

#### Distribution

*Trichomycterus ytororo* is so far known only from its type locality, the Tabay waterfalls in the Tabay stream (Fig 5), a tributary of the left bank of the Paraná River, province of Misiones, northeast of Argentina (Fig 4).

#### Etymology

The specific epithet *ytororo* derived from the indigenous language Guaraní (“ytororõ”) which means “waterfall” in reference to the habitat occupied by the new species. A noun in apposition.

## Discussion

The new species proposed herein is allocated in the genus *Trichomycterus* (subfamily Trichomycterinae) due to the presence of all the synapomorphies for the clade that includes all the trichomycterids excepting the Copionodontinae and Trichogeninae and lacks those derived characters that support the clade comprising the subfamilies Glanapteryginae, Sarcoglanidinae, Stegophilinae, Tridentinae, and Vandelliinae [3]. The presence of the *levator internus 4* muscle originating from the neurocranial floor and the dorsal face of the posttemporo-supracleithrum, a synapomorphy for Trichomycterinae *latu sensu* [13], in the new taxon also supports its inclusion in the subfamily. Among the Trichomycterinae, the new taxon also lacks the diagnostic characters of *Bullockia*, *Eremophilus*, *Hatcheria*, *Ituglanis*, *Rhizosomichthys*, *Scleronema*, and *Silvinichthys* [3,4, 18,19,20,21,22,23]. In view of these facts, the inclusion of the new species in the genus *Trichomycterus* is the most plausible action for the moment.

The non-monophyletic nature of the group and the long and complicated taxonomic history has placed *Trichomycterus* the most formidable problem in the systematics of the Tricomycteridae [3]. Many species of *Trichomycterus* have uninformative descriptions and are associated to old type material, limiting the applicability of the name only to the types [24]. Currently, the genus contains over 170 species [6], however, its known diversity remains increasing along the recent years. Even with these obstacles, species groups within *Trichomycterus* were defined based on morphological characteristics [11,25,26,27,28,29,30,31], some of them constantly redefined and re-diagnosed, such as the *Trichomycterus brasiliensis* species complex [14,32,33], which, to date, its monophyly could not be consistently assessed [34]. Besides, these characters and all species included on these groups were not placed into a comprehensive phylogenetic analysis, therefore with the possibility of representing artificial groups due to possible parallelisms.

The external and internal characters present in *T*. *ytororo* do not fit with any of the previous suggested species complexes with the exception of the group comprising *T*. *crassicaudatus*, *T*. *igobi*, and *T*. *stawiarski*. This group was proposed based on the presence of the procurrent caudal-fin rays thickly ossified and rigid with a slender distal tip; the dorsal procurrent caudal-fin rays extending along the tips of at least 10 neural spines; and 10–11 branchiostegal rays [11] The two first conditions are shared with in *T*. *ytororo* (Table 1, Fig 3) and contrast with the procurrent caudal–fin rays thin and flexible [11,35] extending usually for seven or eight neural spines tips, as observed in other species within the genus [11]. In addition, the species of the *Trichomycterus stawiarski* group have numerous procurrent dorsal caudal-fin rays (24 or more), in comparison with other congeners (Table 1, Fig 3). Although the number of the caudal–fin procurrent rays are not available for all species, the closest value was observed in *T*. *steindachneri* DoNascimiento, Prada-Pedreros & Guerrero-Kommritz [24]

Besides these osteological traits, the four species show a similar color pattern consisting of two distinct layers of pigmentation: deep layer with large black blotches irregular in shape and size, overlapped by a superficial layer of small and less pigmented spots [11,35] (Figs 1 and 2). In addition, the blotches are more concentrated on the dorsum of the trunk, becoming more scattered towards ventral region. However, considering the high variation of the color pattern within the genus, only an analysis including an extensive comparative material of *Trichomcyterus* and other Trichomycterinae in a phylogenetic context could provide an adequate framework for interpreting these characteristics of coloration. *Trichomycterus ytororo* has the *extensor tentaculi* muscle originating on both suspensorium and neurocranium (Table 1), trait only observed in *T*. *stawiarski* and *T*. *davisi* within the genus [13,36]. At the same time, myological data for *T*. *crassicaudatus* and *T*. *igobi* and the most species on genus are not available. As consequence, the origin of this character remains uncertain considering the well-supported relationship among *T*. *crassicaudatus*, *T*. *igobi* and *T*. *stawiarski* [36].

The putative monophyly of the clade formed by *T*. *crassicaudatus*, + *T*. *igobi*, + *T*. *stawiarski* [11] would be also supported by the presence of 10–11 branchiostegal rays, contrasting with other trichomycterids which have usually 5–8 branchiostegal rays, a plesiomorphic condition [11]. Based on this continuous character (10–11 branchiostegal rays), the recently described species, *T*. *perkos*, was proposed as possible sister taxon of this clade [36]. *Trichomycterus ytororo* has nine branchiostegal rays, a condition not present *T*. *igobi*, *T*. *crassicaudatus* and *T*. *stawiarski* also unusual among trichomycterids [35]

Even so, the aforementioned presumably derived character states shared by the species herein proposed as belonging to the *Trichomycterus stawiarski* group (Table 1) suggest their close relationship and the reduced number of branchiostegal rays in *T*. *ytororo* could be interpreted as a homoplastic feature. However, a comprehensive phylogenetic analysis of the genus is still needed to assess if those character states are more parsimoniously attributed to common ancestry or parallelisms and to test the monophyly of this putative clade.

*Trichomycterus crassicaudatus*, *T*. *igobi*, and *T stawiarski* are endemic to the Iguazú (or Iguaçu, in Brazil) River basin, a left bank tributary of the Paraná River, while *T*. *ytororo* inhabits a downstream section of the same basin (Fig 5). Curiously, all these species are known from few collecting sites or only from their type localities (as *T*. *ytororo*), possibly indicating some ecological constraints in their distributions.

## Supporting information

S1 Appendix List of material examined. (PDF)

S2 Appendix With additional morphometric and meristic tables. (PDF)
