# Supplementary material for: Living in the waterfalls: A new species of Trichomycterus (Siluriformes: Trichomycteridae) from Tabay stream, Misiones, Argentina
Source: PLoS One. 2017 Jun 22;12(6):e0179594. doi: 10.1371/journal.pone.0179594 (PMC5480901; doi:10.1371/journal.pone.0179594)
Supplement: S1 Appendix — (PDF) [file pone.0179594.s001.pdf]

Comparative material.

Institutional abreviations: AMNH, The American Museum of Natural History, New York; BMNH, British Museum of Natural History, London; CI-FML, Colección Ictiologíca Fundación Miguel Lillo; FMNH, Field Museum of Natural History, Chicago; LIRP, Laboratório de Ictiologia de Ribeirão Preto, Ribeirão Preto; MCN, Museu de Ciências Naturais Fundação Zoobotânica do Rio Grande do Sul, Porto Alegre; MCP Museu de Ciências e Tecnologia, Pontifícia Universidade Católica do Rio Grande do Sul, Porto Alegre; MNRJ, Museu Nacional, Rio de Janeiro; MZUSP, Museu de Zoologia, Universidade de São Paulo, São Paulo; UFRGS, Universidade Federal do Rio Grande do Sul, Porto Alegre; UMMZ, University of Michigan Museum of Zoology, Ann Arbor ; USNM, National Museum of Natural History, Smithsonian Institution, Washington.

***Bullockia maldonadoi*:** USNM 84344, 2, paralectotypes of *Hatcheria maldonadoi*, 45.2-49.5 mm SL, Chile, Nonguen River. USNM 399186, 8 (2 c&s), 31.1-55.9 mm SL, Chile, Laja River.

***Eremophilus mutisii*:** USNM 79201, 3 (3 xr), 105.5-165.0 mm SL, Colômbia.

***Hatcheria macraei*:** FMNH 58529, 7 (1 c&s), 20.8-96.8 mm SL, Argentina, San Juan River. AMNH 963, 1, 137.4 mm SL, Argentina, Comajo Creek. UFRGS 17696, 2 (1 c&s), 40.0-50.0 mm SL, Chile, Allipen River.

***Ituglanis apteryx*:** MZUSP 115048, holotype, 62.3 mm SL, Brazil, Curuá River.

***I. australis*:** UFRGS 12578, 1, paratype, 40.2 mm SL, Brazil, Do Pinto Creek; UFRGS 13600, 1, paratype, 75,2 mm SL, Brazil, Santa Isabel Creek.

***I. bambui*:** MZUSP 79860, holotype, 41.7 mm SL, Brazil, Goiás, Angelica Cave.

I. boitata: UFRGS 17617, 5 (1 c&s), paratypes, 43.0-66.2 mm SL, Brazil, Água Parada Creek.

I. cahyensis: MNRJ 28404, holotype, 46.9 mm SL, Brazil, Dos Palmares Creek; MNRJ 32080, 1, 35.6 mm SL, Brazil, Cahy River.

I. eichhorniarum: AMNH 233244, 1, 64.9 mm SL, Bolivia, La Chonta River; AMNH 233230, 2, 42.8-60.1 mm SL, Bolivia, Amboro National Park. MNRJ 780, 2, 34.2 mm SL, paralectotypes of *Trichomycterus eichorniarum*, Brazil, Paraguai River.

I. epikarsticus: MZUSP 79869, 1, Brazil, São Mateus Cave.

I. gracilior: FMNH 53264, holotype of *Pygidium gracilior*, not measured, Guyana, Potaro River basin.

I. herberti: MNRJ 1429, 2, syntypes of *Trichomycterus herberti*, 52.7-54.9 mm SL, Brazil, Bodoquena River; MNRJ 1428, 1, syntype of *Trichomycterus herberti*, 60.5 mm SL Brazil, Bodoquena River.

I. ina: MZUSP 112508, 4, 47.4–57.8 mm SL, Brazil, Carajás.

I. macunaima: MZUSP 88452, holotype, 30.7 mm SL, Brazil, Corixo da Saudade, Araguaia River basin.

I. mambai: MCP 42538, holotype, 53.8 mm SL, Brazil, Lapa do Sumidouro cave; MCP 42537, 1, paratype, 68.9 mm SL, Brazil, Lapa do Sumidouro cave.

I. paraguassuensis: USNM 301016, 5, paratypes, 20.9–37.7 mm SL, Brazil, Paraguaçu River.

I. parahybae: MNRJ 22980, 3, 39.8–45.0 mm SL, Brazil, Safra Creek; FMNH 58576, 1, 29.3 mm SL, Brazil, Parahyba.

I. passensis: MZUSP 79869, holotype, 26.2 mm SL, Brazil, Passa Três Cave; MZUSP 80099, 2, 50.5–54.1 mm SL Brazil, Passa Três Cave.

I. proops: MCP 20620, 8, 60.1-68.3 mm SL, Brazil, Betari River. MZUSP 60255, 10 (2 c&s), 57.5–60.4 mm SL, Brazil, Da Onça Creek. MNRJ 781, 3, paralectotypes of *Trichomycterus proops*, 49.4–63.5 mm SL, Brazil, Ribeira de Iguape River.

I. ramiroi: MZUSP 79865, holotype, 28.1 mm SL, Brazil, São Bernardo Cave.

Scleronema angustirostre: BMNH 1944.6.20.1, 1 (xr), paratype of *Pygidium angustirostris*, 34.5 mm SL. MNRJ 3605, 2 (xr), paratypes of *Pygidium angustirostris*, 19.9-32,4 mm SL.

S. minutum: BMNH 1891.3.16.84-86, 3 (xr), syntypes of *Trichomycterus minutus*, 16.6–32.4 mm SL, Brazil, San Lorenzo District.

S. operculatum: FMNH 58080, holotype, 65.9 mm SL (xr), Brazil, Cacequy; FMNH 58520, paratypes, 3, 53.3–58.6 mm SL, Brazil, Cacequy.

Silvinichthys bortayro: AMNH 233621, 1, paratype, 19.9 mm SL, Argentina, Salta; MZUSP 83359, 1, paratype, 27.7 mm SL, Argentina, artificial well near Arenales River.

S. mendozensis: MZUSP 75189, 1, 44.5 mm SL, Argentina, Salto River; USNM 84558, 7, 30.0–65.8 mm SL, Argentina, Blanco River. USNM 84558, 7 (1 xr), 30.0-65.8 mm SL, Argentina, Blanco River.

Trichomycterus albinotatus: MZUSP 42312, holotype, 45.3 mm SL, Brazil, Preto River.

T. alternatus: FMNH 58083; paratypes of *Pygidium alternatum*, 10 (4 xr), 47.5-66.2 mm SL, Brazil, Doce River.

T. alterus: AMNH 12241, holotype of *Pygidium alterum*, 27.6 mm SL, Argentina, Los Sauces River. CI-FML 2085, 1, 49.4 mm SL, Argentina, La Rioja.

T. areolatus: UFRGS 10792, 4, 33.1-54.4 mm SL, Chile, Província de Cautín, río Allipen; USNM 399180, 10 (2 c&s), 41.4-88.4 mm SL, Chile, Lava River.

T. argos: MZUSP 106274, 3, paratypes, 57.2-92.1 mm SL, Brazil, Serra Nova Creek.

***T. auroguttatus*:** MZUSP 43341, holotype, 49.7 mm SL, Brazil, Marimbondo River; MZUSP 43342, 4, paratypes, 33.6-47.3 mm SL, Brazil, Marimbondo River.

***T. bahianus*:** MZUSP 43340, holotype, 68.0 mm SL, Brazil, unnamed stream tributary to Ribeirão Caveira, Una River basin; MZUSP 38636, 4, paratypes, 44.9-87.3 mm SL, unnamed stream tributary to Ribeirão Caveira, Una River basin.

***T. balios*:** UFRGS 16229, holotype, 82.0 mm SL, Brazil, Santa Cruz River; UFRGS 6831, 14 (2 c&s), paratypes, 27.3-87.5 mm SL, Brazil, Santa Cruz River.

***T. barbouri*:** CI-FML 4742, 63.6-75.6 mm SL, Argentina, Salta, Calchaqui River;

***T. belensis*:** CI-FML 2530, holotype, 63.7 mm SL, Argentina, stream tributary to Laguna Blanca; FML 2531, 3, paratypes, 40.8-59.6 mm SL, Argentina, stream tributary to Laguna Blanca.

***T. borellii*:** MZUSP 2208, 1, 57.8 mm SL, Argentina, Mendoza River;

***T. boylei*:** CI-FML uncatalogued, 3, 38.0-53.3 mm SL, Argentina, creek near Tres Cruces;

***T. brachykechenos*:** MCN 18929, holotype, 61.1 mm SL, Brazil, Dos Sinos River; UFRGS 16244, 1 (c&s), paratype, 51.6 mm SL, Brazil, Dos Sinos River.

***T. brasiliensis*:** LIRP 1968, 3, 50.7–100.7 mm SL, Brazil, Da Prata River basin; FMNH 58078, 11, 42.6-102.4 mm SL, Brazil, Doce River.

***T. candidus*:** LIRP 7425, 3, 37.0–49.3 mm SL, Brazil, Ribeirão Bom Jesus; MNRJ 5209, holotype of *Eremophilus candidus*, 58.5 mm SL, Brazil, Espirito Santo River.

***T. castroi*:** MCP 39099, 1, 48.0 mm SL, Brazil, Do Bugre Creek .MZUSP 36964, holotype, 118.3 mm SL, Brazil, branch of Iguaçu River.

***T. catamarcensis*:** FML 2507, holotype, 36.5 mm SL, Argentina, stream tributary to Laguna Blanca; FML 2508, 4, paratypes, 32.5-35.2 mm SL, Argentina, stream tributary to Laguna Blanca.

T. caudofasciatus: MCP 35030, 2, paratypes, 37.1–38.0 mm SL, Brazil, Caparaó River.

T. concolor: MZUSP 43347, holotype, 62.8 mm SL, Brazil, stream on São Francisco River basin;

T. corduvensis: FML 2463, 2, 57.7-79.5 mm SL, Argentina, Protero River.

T. crassicaudatus: MZUSP 88518, holotype, 109.2 mm SL, Brazil, Jordão River.

T. cubataonis: MNRJ 12490, holotype, 47.1 mm SL, Brazil, Cubatão River.

T. dali: MZUSP 106635, 1, paratype, 47.3 mm SL, Brazil, Saracura Cave.

T. davisi: FMNH 60309, holotype, 43.8 mm SL (xr), Brazil, Serrinha, Iguaçu River basin. FMNH 52242, 8 (4 xr), paratypes, 20.9-44.5 mm SL, Brazil, Serrinha, Iguaçu River basin; LIRP 4962, 2, 24.1–29.7 mm SL, Brazil, Jordão River.

T. diabolus: MZUSP 78860, holotype, 53.7 mm SL, Brazil, São Carlos Creek.

T. diatropoporus: MCP 46947, holotype, 58.8 mm SL, Brazil, Da Prata River; MCP 40933, 2 (c&s), paratypes, 38.8-39.7 mm SL, Brazil, Da Prata River.

T. emanueli: UMMZ 141936, 3, paratypes of *Pygidium emanueli emanueli*, 101.6-117.5 mm SL, Venezuela, Chama River.

T. giganteus: MCP 35028, 3, paratypes, 106.4–130.1 mm SL, Brazil, Guandu–Mirim River.

T. gorgona: ANSP 149946, holotype, 64.8 mm SL, Colombia, freshwater stream at Gorgona Island.

T. guianense: MZUSP 109099, 4, 62.7-85.0 mm SL, Guyana, Kuribrong River.

T. hasemani: ANSP 175851, 10 (2 c&s), 13.0-13.5 mm S, Guyana, unnamed blackwater creek.

T. hualco: CI-FML 2601, holotype, 68.3 mm SL, Argentina, Hualco River.

T. igobi: MZUSP 94843, 3, paratypes, 82.3-88.9 mm SL, Brazil, Jordão River.

T. iheringii: FMNH 58074, 2, 134.3-127.6 mm SL, Brazil, Sapina; LIRP 1055, 2, 77.4–88.9 mm SL, Brazil, Bonito River.

T. immaculatus: FMNH 58079, 2, 70.7-81.5 mm SL, Brazil, Doce River. LIRP 285, 2, 82.2–102.3 mm SL, Brazil, Santo Antônio River.

T. itacambirussu: MZUSP 58493, holotype, 70.9 mm SL, Brazil, Do Cabral Creek.

T. itacarambiensis: MCP 34305, 1, 59.4 mm SL, Brazil, Olhos d'Água Cave; MZUSP 42649, holotype, 47.2 mm SL, Brazil, Olhos d'Água Cave.

T. itatiayae: MNRJ 792, lectotype, 67.9 mm SL, Brazil, Ribeirão da Tapera.

T. jequitinhonhae: MZUSP 58497, holotype, 70.5 mm SL, Brazil, Laranjeiras Creek;

T. johnsoni: ANSP 53873, holotype, 12.3 mm SL, Brazil, Descalvados; MZUSP 95013, 2, 13.1-14.5 mm SL, Brazil, Mutum River.

T. landinga: MZUSP 58496, holotype, 43.3 mm SL, Brazil, Moquém Creek.

T. longibarbatus: MZUSP 43339, holotype, 57.9 mm SL, Brazil, Santa Tereza Village.

T. maracaiboensis: UMMZ 142484, 1, paratype of *Pygidium banneaui maracaiboensis*, 37.0 mm SL, Venezuela, Machango River.

T. maracaya: MCP 34575, 2, paratypes, 30.7–31.7 mm SL, Brazil, Pardo River basin.

T. mboycy: MZUSP 94956, 1, 68.6 mm SL, Brazil, Jordão River.

T. mimonha: MCP 18021, 2, 46.3–67.6 mm SL, Piquete River. MZUSP 43343, holotype, 56.2 mm SL, Brazil, Benfica River.

T. mirissumba: MZUSP 43345, holotype, 57.7 mm SL, Brazil, Preto River.

T. naipi: UFRGS 11405, 4, 55.6-65.6 mm SL, Brazil, Passo do Pano Creek.

T. pantherinus: MCP 35029, 2, paratypes, 39.6–41.9 mm SL, Brazil, Da Prata River.

T. paolence: MZUSP 108930, 3, 51.8-83.9 mm SL, Brazil, unnamed stream tributary to Guarapiranga reservoir.

T. paquequerense**:** MZUSP 53755, 4, 25.2-61.5 mm SL, Brazil, Ribeirão dos Andradas Creek.

T. payaya**:** MNRJ 36665, holotype, 38.5 mm SL, Brazil, Olhos d'Água Creek.

T. perkos**:** MCP 46701, 1, paratype, 48.9 mm SL, Brazil, unnamed stream tributary to Erechim River; MCP 46711, 1, paratype, 69.1 mm SL, Brazil unnamed stream tributary to Sepultura Creek.

T. plumbeus**:** UFRGS 13947, 1, 62.5 mm SL, unnamed stream of Paraná River basin.

T. poikilos**:** UFRGS 16239, holotype, 63.3 mm SL, Brazil, Tipiaia Creek; UFRGS 16240, 10 (3 c&s), paratypes, 33.1-66.8 mm SL, Brazil, Tipiaia Creek.

T. potschi**:** MCP 29061, holotype, 78.6 mm SL, Brazil, Das Cachoeiras River.

T. pradensis**:** MNRJ 28483, holotype, 64.2 mm SL, Brazil, Jucuruçu River; MNRJ 28484, 8, paratypes, 36,7-110,5 mm SL, Brazil, Jucuruçu River.

T. pseudosilvinichthys**:** FML 2588, holotype, 60.6 mm SL, Argentina, Amarillo River.

T. ramosus**:** CI-FML 2070, holotype, 59.0 mm SL, Argentina, Laguna Blanca; FML 2071, 5, paratypes, 60.2-64.3 mm SL, Argentina, Laguna Blanc.;

T. reinhardti**:** FMNH 58081, 1, 53,7 mm SL, Brazil, Itabira River; MZUSP 94511, 4, 47.7-70.14 mm SL, Brazil, Itabira River.

T. riojanus**:** MACN 5175, holotype of *Pygidium riojanum*, 60.6 mm SL, Argentina, unnamed stream at La Rioja.

T. rivulatus**:** ANSP 22004, holotype of *Trichomycterus pardus*, 59.5 mm SL, Peru, Jequetepeque.

T. roigi**:** CI-FML 1503, 2, 51.1-61.4 mm SL, Argentina, unnamed stream north of Orosmayo.

T. romeroi**:** ANSP 69331, holotype of *Pygidium romeroi*, 54.9 mm SL, Colombia, Honda.

***T. santaeritae*:** FMNH 58577, holotype of *Pygidium santaeritae*, 19.6 mm SL, Brazil, Preto River.

***T. sketi*:** ANSP 189652, 1, paratype, 60.8 mm SL, Colombia, Del Indio Cave.

***T. spegazzinii*:** CI-FML 4747, 3, 35.8-89.1 mm SL, Argentina, Calchaquí River; MACN 4925, 5, syntypes *Pygidium spegazzinii*, 33.7-72.8 mm SL, Argentina, Cachi River.

***T. stawiarski*:** MCP 22587, 2, 87.4–94.9 mm SL, Brazil, Cavernoso River basin; LIRP 5088, 2, 52.8-60.0 mm SL, Brazil, reservoir at Jordão River; MNRJ 9739, holotype of *Pygidium stawiarski*, 67.1 mm SL, Brazil, unnamed stream on Iguaçu River basin; UFRGS 18307, 10 (2 c&s), 31.7-49.4 mm SL, Brazil, Das Torres River.

***T. striatus*:** USNM 305351, 3 (c&s), 28.5-69.9 mm SL, Panama, Puturgandi River. USNM 376566, 13, 43.7-78.2 mm SL, Panama, Veraguas.

***T. taczanowskii*:** MZUSP 26031, 1, 100.6 mm SL, Peru, Chiriaco River.

***T. taeniops*:** ANSP 71638, holotype of *Pygidium tenue*, not measured, Peru, Ucayali River basin.

***T. tiraquae*:** ANSP 69126, holotype of *Pygidium tiraquae*, 33.2 mm SL, Bolivia, Cochabamba.

***T. tropeiro*:** MCP 46171, holotype, 82.3 mm SL, Brazil, Das Antas River; UFRGS 8818, 2 (1 c&s), paratypes, 46.8-85.1 mm SL, Brazil, Das Antas River.

***T. tupinamba*:** MZUSP 61686, 2, 50.3-54.8 mm SL, Brazil, Betari River.

***T. uisae*:** ANSP 187498, 1, paratype, 44.2 mm SL, Colombia, El Misterio Cave.

***T. variegatus*:** LIRP 647, 3, 43.1–49.8 mm SL, Brazil, Paraná State, São Francisco River; MZUSP 42316, holotype, 39.8 mm SL, Brazil, Do Peixe River.

***T. vermiculatus*:** MZUSP 87189, 4, 36.1-99.1 mm SL, Brazil, Içara Creek.

T. weyrauchi**:** ANSP 71639, holotype of *Pygidium weyrauchi*, 41.2 mm SL, Peru, Ucayali River basin.

T. yuska**:** FML 2535, holotype, 88.6 mm SL, Argentina, Aguas Calientes Creek.

T. zonatus**:** FMNH 58573, holotype of *Pygidium zonatum* (xr), 53.6 mm SL, Brazil, Água Quente; FMNH 58574, paratypes of *Pygidium zonatum*, 2 (xr), 42.2-48.0 mm SL, Brazil, Água Quente.
