# Supplementary material for: Living in the waterfalls: A new species of Trichomycterus (Siluriformes: Trichomycteridae) from Tabay stream, Misiones, Argentina
Source: PLoS One. 2017 Jun 22;12(6):e0179594. doi: 10.1371/journal.pone.0179594 (PMC5480901; doi:10.1371/journal.pone.0179594)
Supplement: S2 Appendix — (PDF) [file pone.0179594.s002.pdf]

S2 Appendix with additional morphometric and meristic tables.

Table 3. Morphometric data for *Trichomycterus ytororo*: holotype and paratypes (n = 12). Measurements are expressed in percent of standard length or head length.

	1	2	3	4	5	6	7	8	9	10 (holotype)	11	12
Standard length (mm)	67.3	60.9	64.6	79.2	65.3	61.0	79.4	81.2	103.8	94.2	107.3	85.3
Percent of standard length												
Head length	22.5	21.4	21.8	19.7	21.2	21.9	20.5	21.4	20.0	20.1	20.0	20.7
Predorsal length	63.5	60.2	63.9	62.0	62.3	61.7	62.2	62.1	62.0	60.5	61.3	61.4
Prepelvic length	58.5	55.6	57.6	56.3	56.9	56.6	54.9	57.2	55.5	55.2	56.3	56.9
Preanal length	73.8	67.7	73.2	70.9	72.4	71.8	70.5	71.8	70.9	69.9	71.2	71.9
Scapular girdle width	17.6	18.0	17.5	16.6	17.5	18.3	17.4	17.1	17.2	17.2	16.1	17.0
Trunk length	34.7	38.3	39.3	37.3	37.3	37.8	38.3	38.7	39.3	36.6	38.6	38.5
Pectoral-fin length	11.3	11.5	10.2	11.1	12.4	10.2	12.0	11.5	10.5	10.7	11.1	10.2
Pelvic-fin length	9.7	9.0	8.4	8.2	9.3	8.8	8.6	9.2	7.9	8.3	8.4	8.4
Distance between pelvic-fin base and anus	9.2	8.5	8.2	8.3	8.3	9.9	8.7	8.3	9.0	8.9	9.2	10.2
Caudal peduncle length	21.6	20.3	20.6	21.4	20.9	22.4	22.2	21.6	22.6	23.4	22.7	21.2
Caudal peduncle depth	16.8	16.1	16.6	17.0	16.9	17.7	16.9	17.3	18.1	18.2	17.9	15.6
Body depth	19.3	18.7	19.5	19.1	19.5	19.9	19.4	19.3	19.3	19.9	20.4	18.0
Length of dorsal-fin base	11.6	12.1	12.1	11.5	12.3	12.2	12.2	11.8	12.6	12.2	11.3	11.5
Length of anal-fin base	8.5	10.4	9.3	8.4	9.3	8.7	9.0	8.6	8.3	8.9	8.3	9.5
Percent of head length												
Head width	82.3	86.6	87.0	92.5	94.7	85.9	85.2	88.5	91.1	92.7	90.5	84.2
Nasal barbel length	30.4	31.2	31.0	25.4	33.9	30.3	26.1	33.3	22.5	29.2	29.0	28.0
Maxillary barbel length	33.3	33.8	31.4	23.1	30.2	26.5	28.0	33.1	25.7	32.2	31.4	33.6
Rictal barbel length	25.0	30.5	36.6	26.9	36.0	28.3	29.4	29.9	28.1	28.0	28.6	30.5
Snout length	36.5	40.8	42.4	41.6	42.0	36.9	40.9	40.8	40.6	42.4	42.6	41.8
Interorbital	19.1	19.8	22.9	21.4	21.1	22.0	21.0	22.2	23.9	21.2	23.4	24.4
Mouth width	29.7	33.8	31.1	33.5	31.8	26.8	32.3	32.2	34.5	32.4	34.2	30.7
Eye diameter	13.3	14.7	14.3	14.5	16.1	13.5	13.5	12.8	11.3	12.6	11.8	13.4
Supra-orbital pore distance	10.4	12.4	8.6	9.2	6.8	11.9	14.0	11.8	12.2	11.8	8.8	9.0

Table 4. Morphometric data for *Trichomycterus ytororo*: holotype and paratypes (n = 12). Measurements are expressed in mm.

	1	2	3	4	5	6	7	8	9	10 holotype	11.0	12.0
Standard length (mm)	67.3	60.9	64.6	79.2	65.3	61.0	79.4	81.2	103.8	94.2	107.3	85.3
Head length	15.2	13.1	14.1	15.6	13.8	13.4	16.3	17.4	20.8	18.9	21.5	17.7
Predorsal length	42.7	36.7	41.2	49.1	40.7	37.6	49.4	50.4	64.3	57.0	65.7	52.4
Prepelvic length	39.3	33.9	37.2	44.6	37.2	34.5	43.6	46.5	57.6	52.0	60.4	48.6
Preanal length	49.6	41.3	47.3	56.2	47.3	43.8	56.0	58.3	73.6	65.8	76.4	61.3
Scapular girdle width	11.8	11.0	11.3	13.2	11.4	11.1	13.9	13.9	17.8	16.2	17.3	14.5
Trunk length	23.3	23.4	25.4	29.5	24.3	23.1	30.4	31.4	40.8	34.4	41.4	32.9
Pectoral-fin length	7.6	7.0	6.6	8.8	8.1	6.2	9.5	9.3	11.0	10.1	12.0	8.7
Pelvic-fin length	6.5	5.5	5.4	6.5	6.1	5.4	6.9	7.5	8.2	7.8	9.1	7.2
Distance between pelvic-fin base and anus	6.2	5.2	5.3	6.6	5.4	6.0	6.9	6.7	9.3	8.4	9.9	8.7
Caudal peduncle length	14.5	12.4	13.3	16.9	13.7	13.6	17.6	17.6	23.5	22.0	24.3	18.1
Caudal peduncle depth	11.3	9.8	10.7	13.5	11.0	10.8	13.4	14.0	18.8	17.1	19.2	13.3
Body depth	13.0	11.4	12.6	15.2	12.7	12.2	15.4	15.7	20.1	18.8	21.9	15.4
Length of dorsal-fin base	7.8	7.4	7.8	9.1	8.0	7.4	9.7	9.6	13.1	11.5	12.1	9.9
Length of anal-fin base	5.8	6.3	6.0	6.7	6.1	5.3	7.1	7.0	8.6	8.4	8.9	8.1
Head width	12.5	11.3	12.2	14.5	13.1	11.5	13.9	15.4	18.9	17.5	19.5	14.9
Nasal barbel length	4.6	4.1	4.4	4.0	4.7	4.1	4.3	5.8	4.7	5.5	6.2	4.9
Maxillary barbel length	5.0	4.4	4.4	3.6	4.2	3.5	4.6	5.8	5.4	6.1	6.7	5.9
Rictal barbel length	3.8	4.0	5.2	4.2	5.0	3.8	4.8	5.2	5.9	5.3	6.1	5.4
Snout length	5.5	5.3	6.0	6.5	5.8	4.9	6.7	7.1	8.5	8.0	9.1	7.4
Interorbital	2.9	2.6	3.2	3.4	2.9	2.9	3.4	3.9	5.0	4.0	5.0	4.3
Mouth width	4.5	4.4	4.4	5.2	4.4	3.6	5.3	5.6	7.2	6.1	7.3	5.4
Eye diameter	2.0	1.9	2.0	2.3	2.2	1.8	2.2	2.2	2.3	2.4	2.5	2.4
Supra-orbital pore distance	1.4	1.4	1.8	1.4	1.3	0.9	1.9	2.4	2.5	2.3	2.5	1.6

Table 5. Meristic data for *Trichomycterus ytororo*: holotype and paratypes (n = 12).

	1	2	3	4	5	6	7	8	9	10 (holotype)	11	12
Dorsal fin rays	ii,7	ii,7	ii,7	ii,7	ii,7	ii,7	ii,7	ii,7	ii,7	ii,7	ii,7	ii,7
Pectoral fin rays	i,7	i,7	i,7	i,7	i,7	i,7	i,7	i,7	i,7	i,7	i,7	i,7
Pelvic fin rays	i,4	i,4	i,4	i,4	i,4	i,4	i,4	i,4	i,4	i,4	i,4	i,4
Anal fin rays	i,5	i,5	i,5	i,5	i,5	i,5	i,5	i,5	i,5	i,5	i,5	i,5
Rays in the upper hypural plate	i,5	i,5	i,5	i,5	i,5	i,5	i,5	i,5	i,5	i,5	i,5	i,5
Rays in the lower hypural plate	i,7	i,6	i,7	i,7	i,6	i,6	i,7	i,7	i,7	i,6	i,6	i,6
Dorsal procurrent caudal-fin rays	35	35	31	34	33	33	33	32	35	35	31	?
Ventral procurrent caudal-fin rays	13	13	13	13	13	13	13	13	13	13	13	?
Vertebrae	38	38	38	38	37	37	37	37	38	38	38	?
Ribs	11	11	12	11	12	11	12	11	12	11	11	?
